# Importance of Glutamate Dehydrogenase (GDH) in *Clostridium difficile* Colonization *In Vivo*

**DOI:** 10.1371/journal.pone.0160107

**Published:** 2016-07-28

**Authors:** Brintha Parasumanna Girinathan, Sterling Braun, Apoorva Reddy Sirigireddy, Jose Espinola Lopez, Revathi Govind

**Affiliations:** Division of Biology, Kansas State University, Manhattan, Kansas, 66502, United States of America; Universidad Andres Bello, CHILE

## Abstract

*Clostridium difficile* is the principal cause of antibiotic-associated diarrhea. Major metabolic requirements for colonization and expansion of *C*. *difficile* after microbiota disturbance have not been fully determined. In this study, we show that glutamate utilization is important for *C*. *difficile* to establish itself in the animal gut. When the *gluD* gene, which codes for glutamate dehydrogenase (GDH), was disrupted, the mutant *C*. *difficile* was unable to colonize and cause disease in a hamster model. Further, from the complementation experiment it appears that extracellular GDH may be playing a role in promoting *C*. *difficile* colonization and disease progression. Quantification of free amino acids in the hamster gut during *C*. *difficile* infection showed that glutamate is among preferred amino acids utilized by *C*. *difficile* during its expansion. This study provides evidence of the importance of glutamate metabolism for *C*. *difficile* pathogenesis.

## Introduction

*Clostridium difficile*, a major nosocomial pathogen, is the principal causative agent of antibiotic-associated diarrhea and pseudomembranous colitis [1,2,3]. Antibiotic use is the primary risk factor for development of *C*. *difficile* infection because it disrupts the normal protective gut flora and enables *C*. *difficile* to colonize the colon [4]. *C*. *difficile* spores are typically ingested and germinate inside the host. Bile salts, such as taurocholate, are known to induce germination of *C*. *difficile* spores in the gut [5]. Once germinated, the outgrowing vegetative cells colonize the gut where they eventually produce toxins A and B, virulence factors that damage the intestinal tissues resulting in *C*. *difficile* infection [6,7]. *In vitro* studies of *C*. *difficile* have demonstrated that transcription of toxin genes is tightly linked with various bacterial metabolic regulatory networks, and is activated in response to various nutritional signals [8,9,10,11,12,13,14,15]. Here, we sought to determine the metabolic requirements for multiplication and colonization of *C*. *difficile* in the host gut.

Studies on metabolism and nutritional requirements of *C*. *difficile* have indicated that it can ferment free amino acids, and the preferred substrates are low molecular weight peptides [16,17]. Since glutamate is central in amino acid metabolism, and biosynthesis of many other amino acids depend on glutamate, we hypothesized that the ability of *C*. *difficile* to colonize the gut might be dependent on the glutamate utilization pathway. Glutamate dehydrogenases (GDH) are a broadly distributed group of enzymes [18,19] that catalyze the oxidative deamination of glutamate to α-ketoglutarate and ammonia (Glutamate + NAD^+^ + H_2_O αKG + NADH + H^+^ + NH_4_^+^). Some GDH enzymes also catalyze a reverse reaction that generates glutamate by condensation of ammonia and α-ketoglutarate. The physiological role of GDH as either an anabolic or catabolic enzyme is determined by its cofactor specificity (NAD or NADH, NADP or NADPH). In *C*. *difficile*, the GDH enzyme is NAD-specific, and mediates the oxidative deamination of glutamate to produce α-ketoglutarate and ammonia [20]. Each of these products plays important roles in amino acid metabolism in all organisms.

GDH specific Enzyme Immuno Assays (EIA) for the detection of *C*. *difficile* are commercially available. Detection of *C*. *difficile* is currently performed as a two-step process. An ELISA for *C*. *difficile* GDH is performed first, and GDH-positive specimens are tested further for toxin production by ELISA [21,22]. The effectiveness of GDH as a diagnostic marker is well-documented [21,22]. However, the importance of GDH for *C*. *difficile* pathogenesis is not known [21,22]. We previously created a GDH (*gluD*) mutant in *C*. *difficile* JIR8094 strain, and found that the mutant was more sensitive to hydrogen peroxide than the parent strain [23]. We have also shown that *C*. *difficile*-derived GDH can be detected extracellularly [23]. Here, we investigated the importance of GDH in colonization and pathogenesis of *C*. *difficile*. In a hamster model, we found that *C*. *difficile* GDH (*gluD*) mutant failed to colonize the animals or to cause disease. Furthermore, our results indicated that extracellular GDH from *C*. *difficile* might play a role in supporting bacterial colonization in the gut. This is the first time its been demonstrated that a specific amino acid metabolic pathway is essential for *C*. *difficile* pathogenesis.

## Materials and Methods

### Bacterial strains and growth conditions

*C*. *difficile* strains, JIR8094 [24] and the JIR8094:: *gluD* mutant (Table 1), *Clostridium sordellii* strain ATCC 9084 [25] and *Clostridium perfringens* strain SM125 [26] were grown anaerobically (10% H_2_, 10% CO_2_ and 80% N_2_) in TY broth or TY agar as described previously [23,25,27]. *Bacillus subtilis* strain 168 was grown aerobically in LB medium [28]. *E*. *coli* strain S17-1 [29] used for conjugation was cultured aerobically in LB medium supplemented as needed for selection with chloramphenicol (30 μg ml^-1^) or ampicillin (100 μg ml^-1^). All routine cloning and plasmid constructions (Table 1) were carried out using standard procedures. Oligonucleotides used in this study are listed in S1 Table.

**Table 1:** Bacterial strains/ plasmids used in this study.

Strain/ Plasmids	Description	Sources
JIR8094	Erythromycin sensitive derivate of *C*. *difficile* 630 strain	[24]
JIR8094-*gluD* mutant	*C*. *difficile* JIR8094 with intron insertion within *gluD* gene	[23]
DH5α	*E*.*coli* strain—endA1 recA1 deoR hsdR17 (r_K_^-^ m_K_^+^)	NEB labs
S17-1	*E*.*coli* strain–favors conjugation	[29]
SM125	*Clostridium perfringens*	[26]
BS168	*Bacillus subtilis*	[28]
ATCC9084	*Clostridium sordellii*	[25]
pMTL007E5:Cdi-*gluD*-324a	pMTL007C-E5 carrying *gluD* specific intron	This study
pMTL84151	Shuttle vector for *C*. *difficile*	[63]
pRGL51	*gluD* promoter (840 bps of *gluD* upstream) cloned in pMTL84151	[23].
pRGL315	*Clostridium sordellii gluD* in pRG51	This study
pRGL316	*Clostridium perfringens gluD* in pRG51	This study
pRGL75	*Bacillus subtilis rocG* in pRG51	This study
pRGL58	*C*. *difficile gluD* in pRG51	[23]
pRGL77	Modified *C*. *difficile gluD* in pRG51 to express GDH without 20 N terminal amino acids (GDH-20N)	This study
pRGL164	Modified *C*. *difficile gluD* in pRG51 to express GDH without 20 C terminal amino acids (GDH-20C)	This study
*gluD* mutant +pMTL84151	*gluD* mutant with vector pMTL84151	This study
*gluD* mutant + pRGL51	*gluD* mutant expressing *C*. *difficile* GDH	[23]
*gluD* mutant+ pRGL315	*gluD* mutant expressing *C*. *sordellii* GDH	This study
*gluD* mutant+ pRGL316	*gluD* mutant expressing *C*. *perfringens* GDH	This study
*gluD* mutant +pRGL77	*gluD* mutant expressing GDH -20N	This study
*gluD* mutant+ pRGL164	*gluD* mutant expressing GDH -20C	This study
JIR8094+ pMTL84151	Wild type *C*. *difficile* with vector pMTL84151	This study

### Complementation of *C*. *difficile gluD* mutant with GDH coding regions of different bacterial sources

The *gluD* mutant complemented was with *C*. *difficile gluD* as described in our earlier study [23]. Briefly, the *gluD* ORF with its upstream regions (840 bps) along with its ribosomal binding site was PCR amplified from JIR8094 chromosomal DNA, using primers gluDP(F) and gluDP(R) (S1 Table), which carried restriction sites *HindIII* and *XbaI* respectively. The resulted PCR product was digested with *HindIII* and *XbaI* and was cloned into pMTL84151 digested with the same to yield pRG51. The *gluD* ORF was PCR amplified from JIR8094 chromosomal DNA using primers ORG72 (with *KpnI*) and ORG79 (with *SacI*) and the PCR product was digested with *KpnI* and *SacI* and cloned into the pRGL51 to construct plasmid pRGL58, where the *gluD* was expressed from its native promoter. Similarly, the *gluD* homologues from *C*. *perfringens*, *C*. *sordellii* and *B*. *subtilis* were PCR amplified from their chromosomal DNA using primers with *KpnI* and *SacI* (S1 Table) and cloned into pRG51 to construct plasmids pRGL316, pRGL315 and pRGL75 respectively, where they were expressed from the *C*. *difficile gluD* promoter. To express *C*. *difficile* GDH without the last 20 C terminal amino acids (*gluD*-20C), primers ORG72 and ORG303 were used to construct pRGL164. To express *C*. *difficile* GDH without the first 20 N terminal amino acids (*gluD*-20N), primers ORG361 and ORG79 were used to construct pRGL79. The GDH expressing plasmids and the vector pMTL84151 were introduced into JIR8094 and *gluD* mutant *C*. *difficile* strains by conjugation [23,25]. Transconjugants carrying different *gluD* constructs or the vector pMTL84151 were grown overnight in TY medium supplemented with thiamphenicol. 10 ml of fresh cultures were inoculated with 100μl of overnight cultures and were grown for 6 hours in TY medium with thiamphenicol. Bacterial cells and the culture supernatants were harvested for the detection of GDH. Accession numbers for *C*. *perfringens gluD*, *C*. *sordellii gluD* and *B*. *subtilis rocG* genes are ABG85534.1, EPZ61548.1 and NP_391659.2, respectively.

### GDH Zymogram and ELISA

Culture supernatants and the cytosolic proteins collected from various GDH expressing bacterial cultures were subjected to GDH in gel activity analysis following the protocol described by Okwumabua *et al*. [30]. The culture supernatants were concentrated 10 folds using the Amicon 8000 series stirred cell fitted with an ultra-filtration membrane with molecular weight cut off range of 10 kDa. The concentrated supernatant and cytosolic extracts were subjected to non-denaturing polyacrylamide gel electrophoresis (PAGE). The samples were resuspended in sample buffer devoid of any denaturing agents and were separated in non-SDS polyacrylamide gels. Electrophoresis was performed in tris-glycine buffer without SDS at 50 Volts. Proteins with NAD specific GDH activity were visualized by immersing the gels in 20ml of 20 mM Tris HCL (pH8.0) reaction buffer with, Nitro Blue Tetrazolium, 0.3 mg/ml; phenazine methosulfate, 0.05 mg/ml. To detect NAD or NADH specific GDH activity, either L-glutamate with 0.5 mM NAD or 50 mM alpha ketoglutarate with 1 mM NADH were added to the reaction buffer respectively. GDH activity could be detected as the purple colored bands in the gels. The presence of *C*. *difficile* GDH in the cecal contents was detected with a commercially available ELISA kit (CDiff Check ™- 60, TechLab Inc., Blacksburg, Va.) in accordance with the manufacturer's instructions.

### Hamster model

Male Syrian golden hamsters (100–120 g) were used for *C*. *difficile* infection. They were housed individually in sterile cages with *ad libitum* access to food and water for the duration of the study. In some experiments when hamsters were challenged with *C*. *difficile* carrying a plasmid constructs, thiamphenicol was given to the hamsters through their drinking water at a concentration of 30 mg per liter to select for retention of the plasmid. Fecal pellets were collected from all hamsters, homogenized in 1 ml saline, and examined for *C*. *difficile* by plating on CCFA-TA (Cycloserine Cefoxitin Fructose Agar- 0.1% Taurocholate) to ensure that the animals did not harbor indigenous *C*. *difficile*. After this initial screening for *C*. *difficile*, hamsters were gavaged with 30 mg/kg clindamycin [31,32]. Vegetative *C*. *difficile* cells were used to infect the hamsters. To standardize the preparation of the bacterial inoculums, 100 μl of an overnight culture was inoculated into 10 ml TY broth medium and grown for 12 hours. A 1 ml sample of the exponentially growing culture was washed once with sterile PBS. The absorbance was then adjusted to 1.0 at OD600 nm. Serial dilutions prepared in sterile PBS were used to enumerate bacterial cell counts. Inoculums were prepared as a 200 μL sample standardized to contain approximately 2000 bacterial cells. Inocula were prepared immediately prior to challenge. The inoculums needed to infect each animal were transported in an independent 1.5 ml Eppendorf tube to the vivarium using the Remel AnaeroPack^TM^ system (one box for each strain) to maintain viability. Immediately before and after infecting the animal a 10 μL sample of the inoculum was plated onto TY with cefoxitine agar to confirm the bacterial count and viability. Seven animals per strain were used for the infection. In each experiment, 4 animals were used as uninfected controls, and received only antibiotics and sterile PBS. Infection was initiated 4 days after clindamycin administration by gavage with 2,000 vegetative cells. Animals were monitored for signs of disease (lethargy, poor fur coat, sunken eyes, hunched posture, and wet tail) every four hours (six times per day) throughout the study period. Hamsters were scored from 1 to 5 for the signs mentioned above (1-normal and 5-severe). Fresh fecal pellets were collected daily from every animal to monitor *C*. *difficile* colonization (see the supporting information) until they began developing diarrheal symptoms. Hamsters showing signs of severe disease (a cumulative score of 12 or above) were euthanized by CO_2_ asphyxiation. Surviving hamsters were euthanized 15 days after *C*. *difficile* infection. Thoracotomy was performed as a secondary mean of death and the cecal samples (contents and tissues) were collected for further analysis. H&E (Hematoxylin and Eosin) staining of cecal tissues were performed. The data were graphed as Kaplan-Meier survival analyses, and compared for statistical significance using the log-rank test using GraphPad Prism 6 software (GraphPad Software, San Diego, CA). All animal studies were conducted with prior approval from the Kansas State University’s Institutional Animal Care and Use Committee.

### Histology and inflammation scoring

The ceca were removed and opened longitudinally, and washed in PBS. Full-thickness sections were fixed in formalin, paraffin embedded, and stained with hematoxylin and eosin. Severity of enteritis and colitis was graded using the three parameters as published previously: i) epithelial tissue damage; ii) mucosal edema; iii) neutrophil infiltration [33,34]. Trained pathologists at KSU diagnostic lab scored the blinded samples from 1 to 3 to each parameter mentioned above. Total histology score (from 0 to 9) was determined by the sum of all these three parameter scores. Results were expressed as mean ± standard error of the mean (SEM) and were analyzed by using the Prism professional statistics software program (GraphPad, San Diego, CA). Unpaired Student *t* tests were used for intergroup comparisons. *P* values of statistically significant differences are shown in each figure.

### Bacterial load measurement and detection in cecal contents

At sacrifice, cecal contents harvested and were processed as follows. The cecal materials from the uninfected and the hamsters that survived the *C*. *difficile* challenge were thick in their consistence. These materials were resuspended in sterile PBS and were centrifuged (20,000 × *g* for 5 min at 4°C) to collect the supernatants. The cecal materials from the hamsters that came down with *C*. *difficile* disease were watery and were clarified by centrifugation. The supernatants collected after centrifugation were stored at -80°C and were later used for GDH ELISA. One gram of cecal slurry collected after the centrifugation contents were resuspended in sterile 1 ml PBS, serially diluted and were plated on CCFA-TA (Cycloserine Cefoxitin Fructose Agar with 0.1% Taurocholate) to quantify *C*. *difficile*, which appeared as yellow colonies. Results were presented as cfu per gram.

### Amino acid analysis of cecal contents

Cecal contents from hamsters were weighed at necropsy and flash frozen in liquid nitrogen. Samples were sent to the University of Michigan, Metabolomics Resource Core for amino acid analysis. Amino acids were analyzed using the EZ-faast kit from Phenomenex–Torrance, CA following the instructions provided by the manufacturer. Samples were extracted, semi-purified, derivatized by a proprietary method, and analyzed by EI-GCMS using internal standards for normalization. Analytes were reported as nM/mg of cecal content. Fold changes between clindamycin treated *vs*. non-antibiotic treated controls, and clindamycin treated *vs*. *C*. *difficile* infected colon samples, were evaluated by Welch's *t*-test; *p*<0.05 was considered to be significant.

## Results

### GDH is important for *C*. *difficile* colonization and infection in hamsters

**Fig 1:**
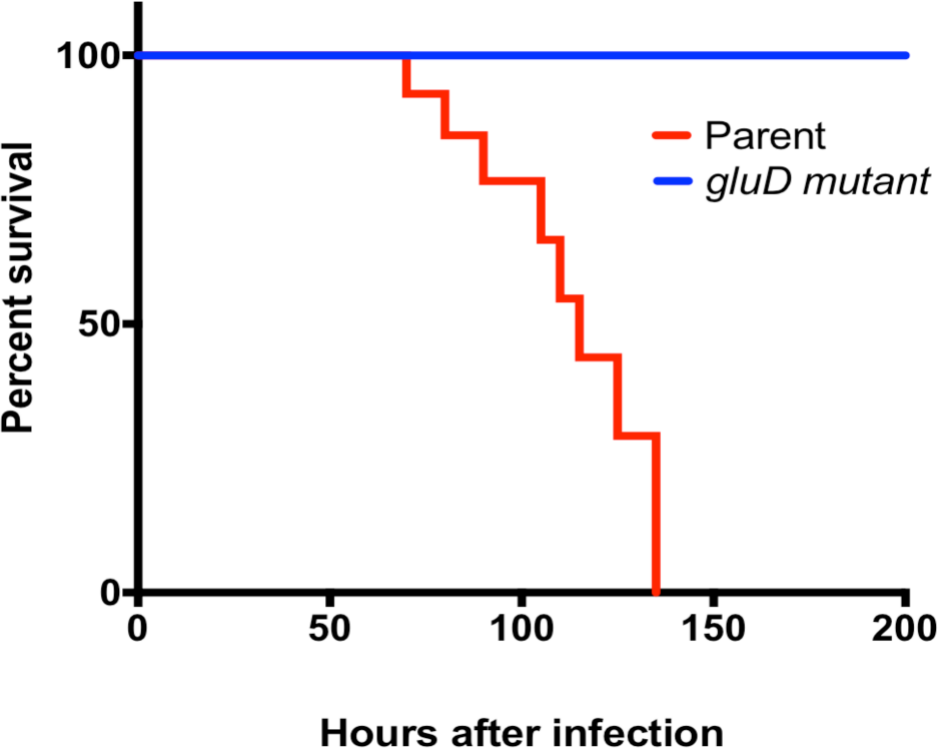
GDH is required for *C*. *difficile* virulence. Kaplan-Meier survival curve of clindamycin-treated Syrian hamsters inoculated with 2,000 vegetative cells of *C*. *difficile* JIR8094 (Parent) or *C*. *difficile* JIR8094::*gluD* (mutant). Animals (n = 7 per group) were monitored every four hours for the symptoms of wet tail, poor fur coat, lethargy, or hunched posture. Moribund animals were euthanized. Log rank statistical analysis was performed; *p* <0.0001.

**Fig 2:**
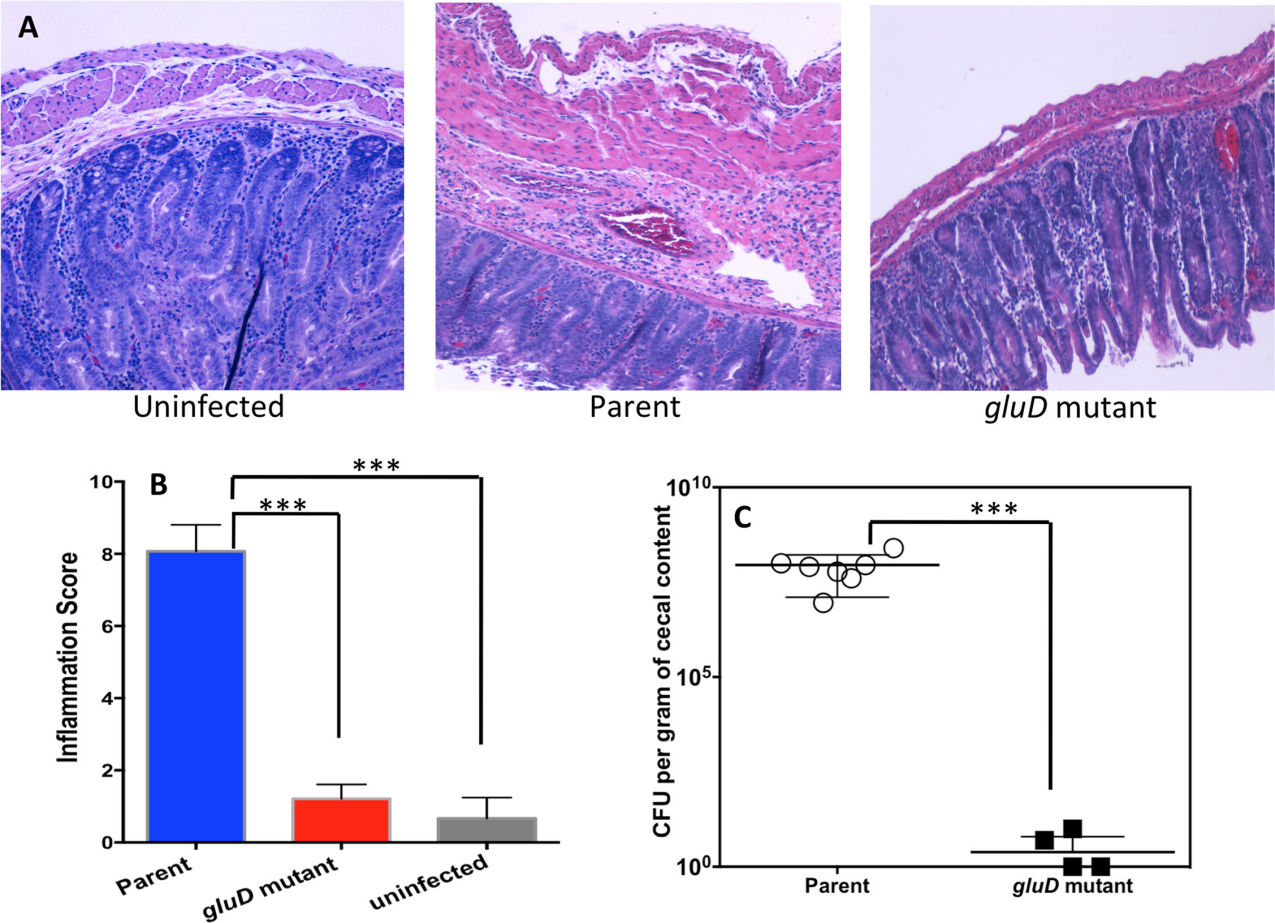
*C*. *difficile* JIR8094::*gluD* mutant does not colonize or induce inflammation in hamsters. **A.** Representative colonic histologic images (hematoxylin and eosin (H&E) staining). Cecal tissues from parental strain-infected hamsters were harvested at the time of sacrifice. Cecal tissues from surviving *gluD* mutant-infected (and uninfected) hamsters were harvested 15 days post-infection. **B.** Histology scores were evaluated as described in the Materials and Methods. **C.** *C*. *difficile* colonization levels for each of the two groups in CFU per gram of cecal content at the time of necropsy. In three of seven *gluD* mutant-infected animals, *C*. *difficile* was not detected and are not represented in the figure (n = 7 per group). Error bars represent SD.

To understand the importance of GDH in *C*. *difficile* pathogenesis, we used a hamster model in which *C*. *difficile* infection is known to cause severe disease symptoms [31]. Syrian male hamsters were gavaged with 2,000 vegetative cells of *C*. *difficile* strain JIR8094 or its *gluD* mutant and monitored for signs of *C*. *difficile* infection. Fecal pellets were collected daily until animals developed diarrheal symptoms; total DNA was extracted and used for qPCR analysis of *C*. *difficile* 16S rRNA (S2A and S2B Table). Results showed that JIR8094 colonized the gut within two days post-inoculation, but the *gluD* mutant either was unable to initiate colonization in hamsters or was rapidly cleared by the host (S2 Table). Animals infected with parental strain JIR8094 succumbed to disease, whereas, the *gluD* mutant was avirulent (Fig 1). Cecal contents from diseased hamsters were collected at sacrifice. Nearly fifteen days post *C*. *difficile* infection, all surviving *gluD* mutant infected hamsters and uninfected control hamsters were sacrificed, and their cecal contents were harvested. The bacterial load in the cecal samples was measured, and cecal tissues were stained with H&E for microscopic evaluation of inflammation (Fig 2A). GDH in the cecal contents was detected using the commercially available ELISA kit specific for *C*. *difficile* GDH. As suspected, GDH could be readily detected in the cecal contents of the JIR8094 infected hamsters but not in cecal contents of the *gluD* mutant (S1 Fig). Little or no inflammation was observed in *gluD* mutant-infected animals; whereas, extensive inflammation accompanied by crypt damage and the influx of inflammatory cells in the lamina propria and sub-mucosa was observed in hamsters infected with the parental strain (Fig 2A). Mean histology scores recorded for parent strain (JIR8094)-treated animals were significantly greater than for *gluD* mutant-treated animals (** *p*<0.005). No or very low inflammation was recorded in *gluD* mutant infected animals (Fig 2B). The cecal contents of JIR8094-infected hamsters contained nearly 10^8^ colony-forming units per gram; however, very few or no *C*. *difficile* cells were detected in the cecal contents of *gluD* mutant-infected animals, suggesting that GDH is needed for *C*. *difficile* colonization and subsequent disease progression in the host gut (Fig 2C).

### *C*. *difficile* is unable to secrete *C*. *difficile* GDH homologues from *B*. *subtilis*, *C*. *sordellii*, and *C*. *perfringens*

In an earlier study, we showed that *C*. *difficile* GDH is secreted from the bacteria during its growth in TY medium [23]. Our finding of GDH in the cecal contents of the JIR8094-infected hamsters suggested that the enzyme might also be secreted during *C*. *difficile* infection in the host. To understand the importance of secreted GDH on *C*. *difficile* pathogenesis, we complemented the *C*. *difficile gluD* mutant with various *gluD* constructs in an attempt to create a *C*. *difficile* strain with non-secreted GDH.

**Fig 3:**
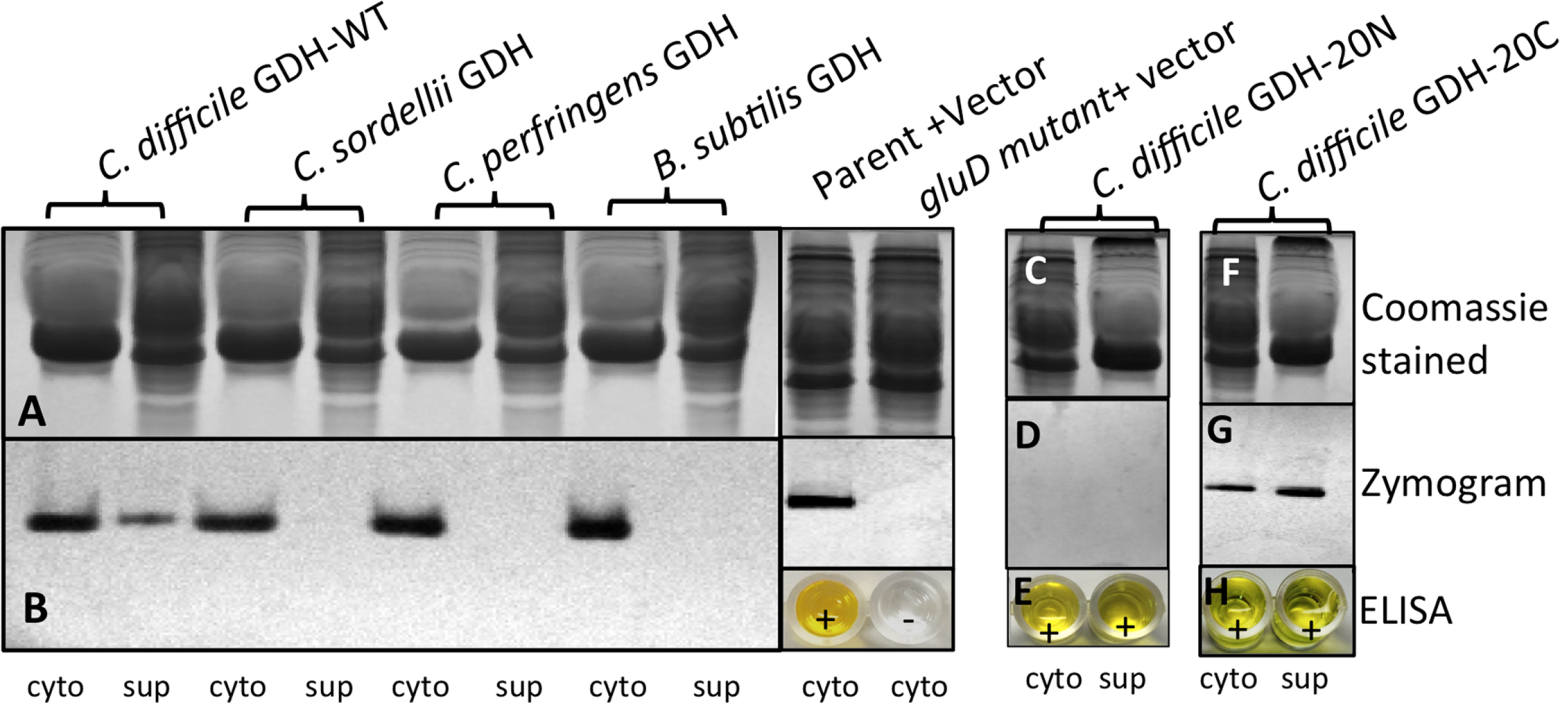
Complementation of *gluD* mutant with various *gluD* constructs. The *gluD* homologues from closely related bacterial species were expressed in the *C*. *difficile gluD* mutant strain, and their secretion from *C*. *difficile* was analyzed. Cytosolic (cyt) and concentrated supernatants (sup) from the bacterial cultures expressing various *gluD* constructs were separated by SDS-PAGE, and were analyzed by Coomassie staining **(A)** and by zymogram **(B)**. *C*. *difficile gluD* constructs with deletions of their N-terminus (panels **C**, **D,** and **E**) or of C-terminus (panels **F**, **G** and **H**) were expressed in *C*. *difficile gluD* mutant, and their secretion from *C*. *difficile* was analyzed by zymogram (**D**&**G**) and ELISA (**E**&**H**).

The first constructs used for complementation were different bacterial *gluD* homologues. NAD-specific GDH encoding genes from *B*. *subtilis*, *C*. *sordellii*, and *C*. *perfringens* were amplified and cloned under the control of the *C*. *difficile gluD* promoter and its ribosome binding site. The resulting constructs were introduced into the *C*. *difficile gluD* mutant, and were tested for the expression and secretion of these non-native GDH enzymes. The *B*. *subtilis*, *C*. *sordellii*, and *C*. *perfringens* GDH enzymes were detected in the cytosolic fractions of the respective transfected *C*. *difficile* cultures. However, none of these GDH enzymes was detected in the culture supernatants (Fig 3A and 3B). This result suggests that *C*. *difficile* GDH is exported out through a specific secretion mechanism in *C*. *difficile*. However, we also recognize that the absence of *C*. *sordellii*, *C*. *perfringens* and *B*. *subtilis* GDH enzymes in the extracellular medium when expressed in *C*. *difficile* may simply due to additional variations associated with their production in this heterologous host. Further characterizations of these strains are currently under progress in our lab.

In bacteria, many secreted proteins require signal peptides to be transported across the cytoplasmic membrane. To assess signal specific secretion of GDH in *C*. *difficile*, we created GDH constructs lacking the 20 amino acids from either N terminal or C terminal part of the enzyme and tested their secretion from *C*. *difficile* cells. Removal of 20 amino acid residues from the N-terminus *C*. *difficile* GDH (GDH-20N) abolished its enzymatic activity (Fig 3C and 3D); however, secretion of GDH was not affected (Fig 3E). Removal of 20 amino acids from the C-terminus of the protein (GDH-20C) did not affect either the enzymatic activity (Fig 3F and 3G) or the secretion of GDH enzyme (Fig 3H).

### Extracellular GDH appears to favor rapid *C*. *difficile* disease progression in hamsters

**Fig 4:**
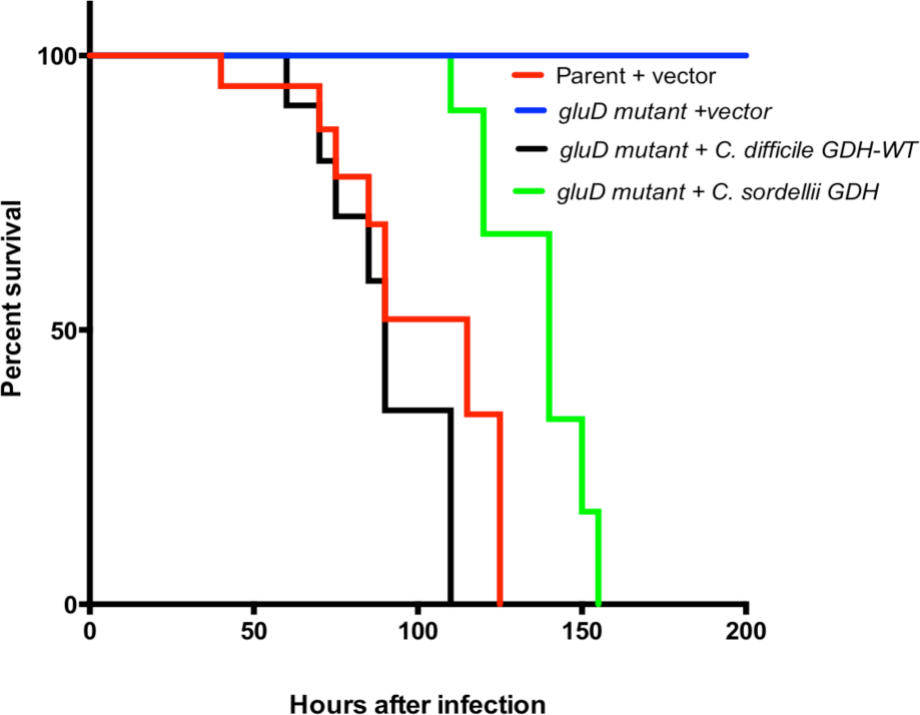
Extracellular GDH enables rapid progression of *C*. *difficile* infection in hamsters. *C*. *difficile gluD* mutant complemented with secretable *C*. *difficile* GDH or with nonsecretable *C*. *sordellii* GDH were used to infect the clindamycin-treated hamsters. Survival rate was plotted using Kaplan-Meier survival curve. Comparisons of *C*. *difficile* GDH-WT vs. *C*. *sordellii* GDH survival curves were made using long rank test; *p* = 0.035.

**Fig 5:**
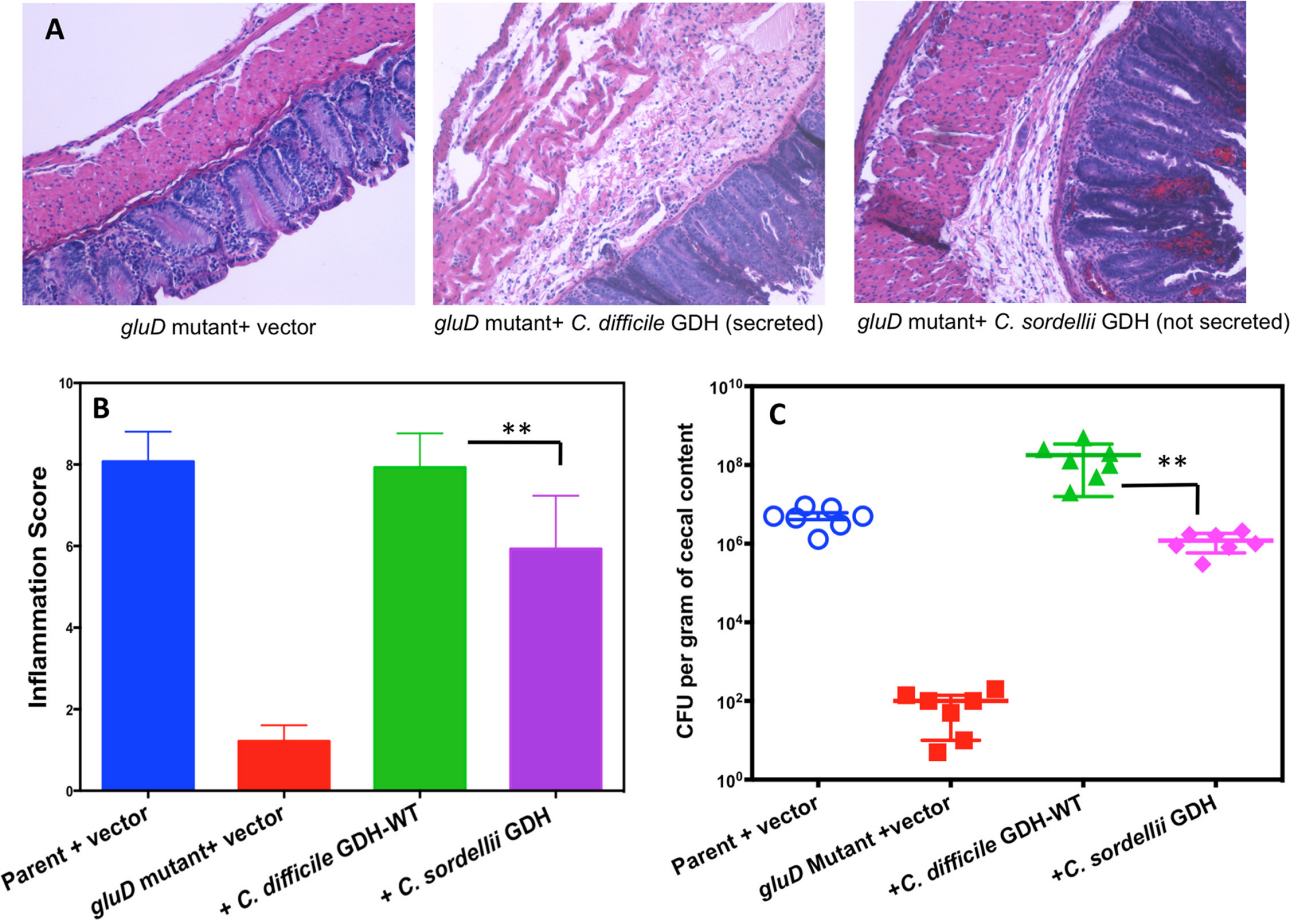
*C*. *difficile gluD* mutant complemented with secretable forms of GDH colonized better and induced more inflammation than the mutant expressing nonsecretable GDH. **A**. Representative image of H&E stained colonic specimens. **B**. Histology scores evaluated as described in the Materials and Methods section. **C**. *C*. *difficile* colonization levels for each of the groups (CFU per gram of cecal content at the time of necropsy). Unpaired *t* test was performed for statistical analysis (n = 7 per group). Error bars represent SD.

Next, hamsters were inoculated with *C*. *difficile gluD* mutant strain complemented with different *gluD* constructs. Initial *in vitro* growth experiments showed that all *C*. *difficile* strains used in this experiment grew at same rate in TY medium (S2 Fig). The strains tested included the *gluD* mutant complemented with nonsecretable-heterologous *C*. *sordellii* GDH, and secretable native *C*. *difficile* GDH. The parental strain and *gluD* mutant carrying vector alone were used as controls. Hamsters were monitored for disease symptoms every four hours, and moribund animals were euthanized. DNA prepared from fecal pellets was subjected to qPCR to monitor *C*. *difficile* colonization. Consistent with our initial experiment, the *gluD* mutant with vector alone failed to colonize the animals or cause disease (Fig 4). The parental strain with vector and the *gluD* mutant complemented with wild-type GDH colonized and caused disease more rapidly than did the *gluD* mutant complemented with the nonsecretable *C*. *sordellii* GDH. The inflammation scores of these groups were also significantly lower than groups that received the parental strain or the *gluD* mutant complemented with secretable *C*. *difficile* GDH (Fig 5A and 5B). Nonetheless, hamsters that were infected with *C*. *difficile* strains that produce heterologous- nonsecretable form of GDH eventually showed signs of infection. *C*. *difficile* was detected in cecal contents and the strains that produce *C*. *sordellii* GDH were able to colonize the gut successfully (Fig 5C). However, fewer *C*. *difficile* cells per gram of cecal content were detected in animals infected with *C*. *difficile* expressing nonsecretable GDH than those expressing secretable forms. This experiment confirmed that GDH was essential for colonization by *C*. *difficile* in the host gut. From these results it also appear that presence of extracellular GDH may favor rapid bacterial colonization.

### Co-infection with the parental strain supports colonization by *gluD* mutant *in vivo*

**Fig 6:**
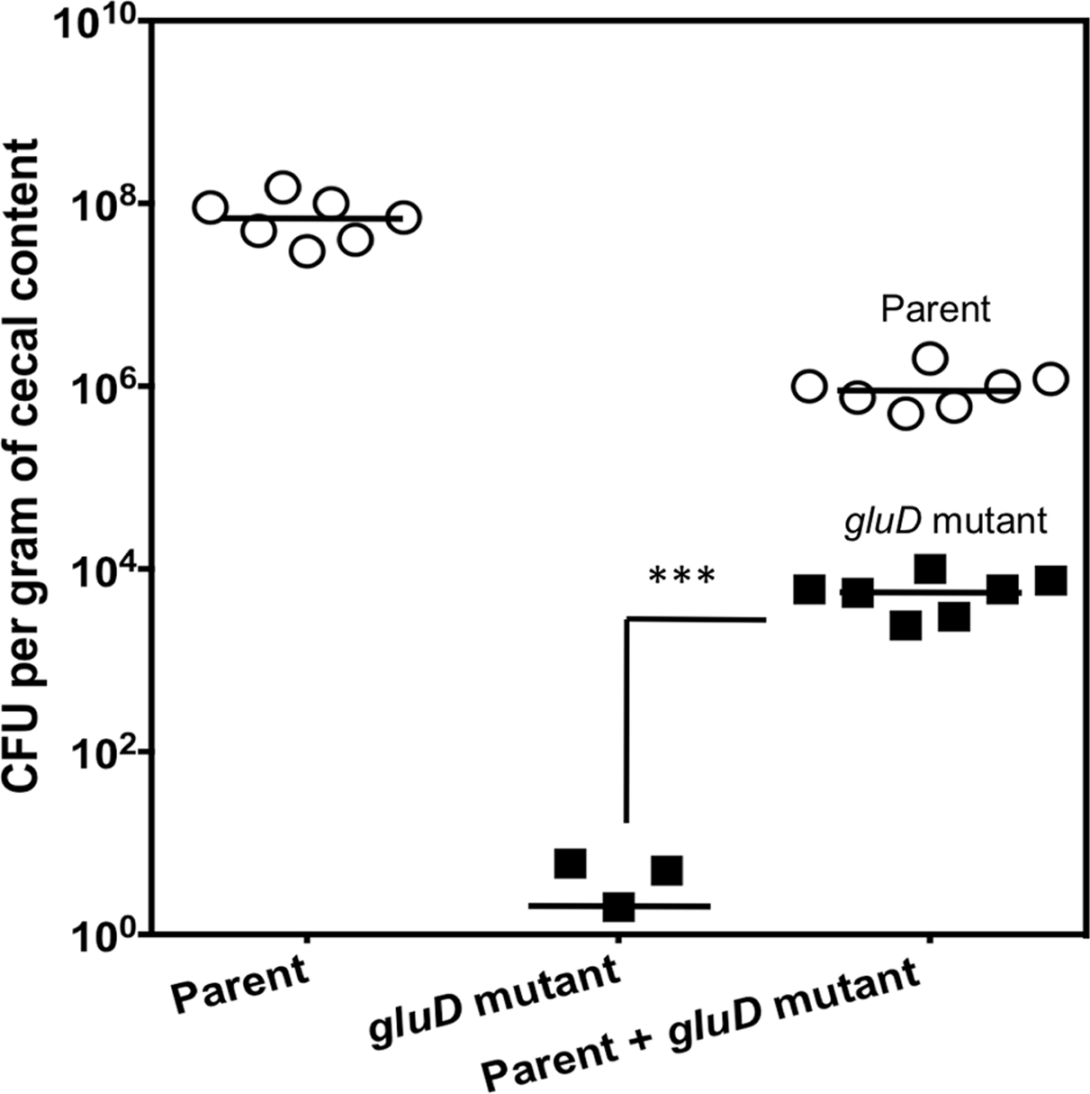
Colonization of hamster gut by *C*. *difficile gluD* mutant in the presence of parental *C*. *difficile* strain. Hamsters were gavaged with a bacterial mixture containing 1000 parent and 1000 *gluD* mutant cells. Bacterial load of each strain at the time of necropsy was measured and presented as CFU per gram of cecal content. Unpaired *t* test was performed for statistical analysis (n = 7 per group). Error bars represent SD.

Hamsters were infected with ∼1000 CFU each of parent and *gluD* mutant cells following clindamycin treatment. The hamsters developed diarrhea at approximately two days post-inoculation, and became moribund at approximately four days post-inoculation (S3 Fig). Cecal contents were harvested, and parental and *gluD* mutant bacterial loads were quantified by plating on CCFA-TA and CCFA-TA with erythromycin. We detected approximately 10^3^ *gluD* mutant bacteria and 10^5^ parental bacteria per gram of cecal content (Fig 6). These results suggested that the *gluD* mutant was able to proliferate to certain extent in the presence of the parental strain. It is likely that extracellular GDH produced by the parental strain or GDH released from the parental strain on lysis supported the growth of *gluD* mutant in the host gut.

### Glutamate in the colon is rapidly utilized by proliferating *C*. *difficile*

It is well established that clindamycin treatment predisposes hamsters to *C*. *difficile* infection [31]. Antibiotic treatments are known to bring dramatic change in the microbial gut community, which alters the metabolic environment within the gut [35,36]. Here, we measured changes in the amino acid pool within the hamster colon following antibiotic treatment and *C*. *difficile* infection.

In this experiment, the first group of hamsters did not receive any treatments and were used as controls (S4 Fig). The second group received clindamycin alone at a concentration of 30 mg/kg weight and was sacrificed six days after antibiotic treatment. The third and fourth groups received clindamycin, and four days later were inoculated with 2000 cells of the parental strain (JIR8094) or *gluD* mutant, respectively. Since we observed colonization of JIR8094 within two days after infection (S2 Table) we chose to sacrifice the *C*. *difficile* infected hamsters two days after gavaging the animals with *C*. *difficile*. Cecal contents were harvested from the sacrificed hamsters, their bacterial load was measured in cecal contents by plating on CCFA-TA; and free amino acid content was quantified as described in Methods (Table 2). Nearly 10^5^ CFU of *C*. *difficile* JIR8094 and 0 to100 CFU of *gluD* mutant cells were detected per gram of cecal contents. Alanine, asparagine, glycine, lysine, isoleucine, methionine, valine, proline, serine, threonine, and ornithine concentrations were increased more than four-fold in clindamycin-treated animals compared to controls. Clindamycin treatment also resulted in two- and three-fold increases in glutamate and glutamine concentrations, respectively. However, glutamate and glutamine concentrations dramatically decreased (30- to 40-fold) after *C*. *difficile* growth in the colon, suggesting that these amino acids were preferentially utilized by *C*. *difficile* in the gut. The data also suggested that ornithine, proline, isoleucine, lysine, serine, and threonine were also used by *C*. *difficile* for growth in hamster colon. The amino acid concentrations in colon contents of hamsters infected with the *gluD* mutant were similar to those in hamsters that received only antibiotics (Table 2). These results reconfirmed our observation that *gluD* mutants do not have the ability to multiply in the hamster gut. These data provide new insight into *C*. *difficile in vivo* metabolism, and suggest that the ability to metabolize amino acids may be important for *in vivo* colonization and subsequent disease progression.

**Table 2:** Amino acid analyses of hamster cecal contents.

Amino Acids	Clindamycin/ No Clindamycin(Positive fold change)	*p* value	Clindamycin/ Clindamycin +*C*. *difficile (Parent strain)*. (Positive fold change)	*p* value	Clindamycin/ Clindamycin + *C*. *difficile (gluD* mutant)(Positive fold change)	*p* value
Alanine	**4.906**	1.02E-04	0.4325	1.78E-03	**3.512**	2.67E-04
Asparagine	**6.45**	2.75E-03	1.21	1.03E-03	**4.51**	1.51E-04
Aspartate	0.249	1.04E-03	0.530	2.05E-04	0.491	1.74E-04
Glutamine	3.39	5.12E-05	**30.28**	1.89E-02	3.12	1.45E-04
Glutamate	2.58	4.13E-04	**46.08**	3.30E-04	2.09	2.35E-03
Glycine	**11.21**	1.12E-03	2.68	6.72E-04	**9.09**	3.62E-04
Histidine	0.36	1.58E-04	0.81	1.37E-03	0.27	2.65E-03
Isoleucine	**6.15**	1.46E-04	**17.90**	1.93E-02	**5.89**	2.36E-04
Leucine	2.88	3.64E-05	2.75	2.98E-03	1.91	2.25E-04
Lysine	**9.92**	1.33E-04	**7.10**	1.96E-03	**8.32**	2.57E-03
Methionine	**6.81**	2.88E-03	2.41	3.97E-03	**6.12**	1.52E-04
Phenylalanine	0.57	2.06E-04	0.68	1.85E-03	0.73	1.96E-03
Proline	**9.11**	1.10E-03	**9.52**	2.45E-04	**8.01**	1.34E-04
Serine	**4.12**	2.92E-04	**10.60**	8.27E-04	**3.98**	1.85E-03
Threonine	**9.85**	4.03E-05	**11.71**	6.56E-04	**8.76**	2.78E-04
Tryptophan	1.65	1.15E-04	3.11	1.98E-04	1.43	1.63E-03
Tyrosine	0.58	1.12E-03	0.29	2.89E-04	0.47	1.59E-03
Valine	**12.67**	4.56E-03	3.30	1.49E-03	**10.21**	1.76E-04
α-Amino isobutyric acid	1.67	2.09E-03	0.562	2.16E-04	1.39	1.70E-04
Ornithine	**41.13**	2.10E-04	**52.05**	1.45E-04	**38.29**	3.42E-03

## Discussion

Glutamate is a key metabolite that serves as a link between carbon and nitrogen metabolism [37]; nearly 88% of cellular nitrogen comes from glutamate [38]. Glutamate metabolism has been shown to be important for the virulence of bacterial pathogens *Staphylococcus aureus* [39], *Neisseria meningitides* [40], and *Helicobacter pylori* [41]. In this study, we found that glutamate utilization is essential for *C*. *difficile* colonization *in vivo* and for subsequent disease progression. The only previously known forms of *C*. *difficile* avirulence in the hamster model were toxin A and B double mutant strains [42,43]. Thus, our finding that *C*. *difficile* depends on free amino acids (especially glutamate) for its colonization and virulence in the host gut is an important observation. The strain JIR8094 is regularly used to create mutants in various genes, including the ones involved in metabolic functions and are used in hamsters for *C*. *difficile* pathogenesis studies [44,45,46,47]. This strain however is non-motile and was shown to produce moderate amount of toxins than the closely related 630∆erm strain [48]. Hence it is possible that deletion of GDH is this background might have attenuated its virulence to greater extent. In a previous study, we showed that a *C*. *difficile gluD* mutant grew more slowly in TY medium than its parent strain during lag phase, but reached a similar growth rate as they approached the logarithmic phase (S2 Fig) suggesting that under *in vitro* growth conditions, the mutant *C*. *difficile* might be using alternate pathways to compensate for the absence of GDH. However, under *in vivo* conditions, the substrates for these alternative pathways may be absent or the gene products of these pathways may not be expressed, resulting in complete cessation of bacterial growth.

Susceptibility to *C*. *difficile* infection following antibiotic treatment in mice has been associated with an increase in the primary bile acid TCA, a germinant of *C*. *difficile* spores, as well as by increases in amino acids, simple sugars, and sugar alcohols—growth substrates for *C*. *difficile* vegetative cells [49]. In another report, investigating the structure and function of the microbiota following fecal microbiota transplantation in patients with recurrent *C*. *difficile* infection, it was found that amino acid transport systems were downregulated following fecal microbiota transplantation [50]. In our study, we measured the free amino acid contents of cecal materials collected from *C*. *difficile*-infected hamsters, and found that *C*. *difficile* preferentially utilizes certain amino acids including glutamate and glutamine. These findings support the idea that amino acid metabolism is a key feature of *C*. *difficile in vivo* colonization. Interestingly, a recent study reported that glutamate was least utilized by *C*. *difficile* when it was grown *in vitro* in a casamino acids-containing medium [51]. The difference in amino acid utilization by *in vitro* grown and *in vivo* grown cells is consistent with greater complexity of *in vivo* metabolic requirements for *C*. *difficile* growth, suggesting that it may be difficult to generate an *in vitro* growth condition that closely mimics the *in vivo* growth requirements.

Typically, bacterial GDH enzymes are cytoplasmic or intracellular membrane-associated proteins. In *C*. *difficile*, GDH was detected both in the cytoplasm and in extracellular culture supernatants. We showed that *C*. *difficile* specifically secreted its own GDH but not GDH enzymes introduced from closely related bacterial species. Using a hamster model, we have provided additional evidence that secreted GDH is important for rapid colonization of *C*. *difficile in vivo*. Glutamate is the precursor of glutathione, a potent antioxidant in intestinal epithelial cells [52]. By scavenging external glutamate in the intestine, *C*. *difficile* may reduce glutathione production by host cells or other microbes, which could help *C*. *difficile* induce increased cellular damage in the intestine thereby facilitating more nutrient release from the host. Glutamate receptors have also been identified in lymphocytes, and were found to influence their ability to modulate immune responses [53]. By scavenging glutamate, an important signaling molecule, *C*. *difficile* may influence a variety of host functions, including the immune response.

In this study, we showed that extracellular GDH improves *C*. *difficile* colonization and disease progression. *C*. *difficile* GDH requires NAD as a co-substrate to metabolize extracellular glutamate in the gut. NAD is present in all living cells, and certain types of cells are known to secrete NAD^+^ and/or respond to NAD^+^ in the extracellular milieu [54,55,56]. NAD from intestinal epithelial cells may be released into the gut lumen when during apoptosis associated with renewal of intestinal epithelia. NAD is an unstable molecule, and it is often difficult to measure its availability in biological samples. We were unsuccessful in our efforts to measure NAD in cecal content of hamsters. However, in a recent report, it was shown that murine colon could release NAD upon nerve stimulation associated with propulsion of gastrointestinal contents [57]. Thus, it is reasonable to assume that sufficient NAD is available to meet the requirements of *C*. *difficile* for extracellular GDH function in the colon. Similar to glutamate, NAD^+^ is also known to acts as a signaling molecule in various cellular functions. In the intestine, extracellular NAD preserves intestinal epithelial barrier function [58]. Thus, by utilizing extracellular NAD, *C*. *difficile* extracellular GDH may enhance toxin-mediated damage to the intestinal barrier in the host.

Since we found that GDH is essential for *C*. *difficile* virulence, it will be interesting to test whether extracellular GDH has a second function. There are many remaining questions: Does GDH act as a glutamate sensor? How does *C*. *difficile* utilize GDH to harvest extracellular glutamate? If extracellular GDH breaks down glutamate to alpha-keto glutarate and ammonia, how are these energy producing molecules transported into *C*. *difficile*? Does extracellular GDH associate with membrane bound transporters of alpha-keto glutarate and ammonia? In *B*. *subtilis* the GlnK enzyme, which is needed for post translational modification of glutamine synthase, is membrane bound and is associated with the ammonia channel Amt [59]. Our BLAST searches for *B*. *subtilis* NrgA (codes for Amt) in *C*. *difficile* genomes did not identify a homologue. Similar BLAST searches for possible alpha-keto glutarate transporters [using the sequence of *Bacillus licheniformis* dicarboxylate transporter- [60]] also didn’t identify a homologue in *C*. *difficile*. It is not clear if these initial failures using *in silico* analyses correctly indicate that these transporters are absent in *C*. *difficile*, or if the *C*. *difficile* transporter is evolutionarily distant.

Finally, we note that community-acquired *C*. *difficile* infections have been on the rise in the past decade [61]. Reasons for the increase in these infections are not yet clear, but a possiblity is suggested by the fact that monosodium glutamate is used extensively as a food preservative [62]. Our study raises the question of whether frequent consumption of monosodium glutamate influences the rate of community-acquired *C*. *difficile* infection. Detailed studies aimed at investigating the role of host nutrient uptake on *C*. *difficile* colonization are needed.

## Supporting Information

S1 Fig GDH ELISA. Detecting GDH in the cecal contents of the hamsters infected with either JIR8094 or *gluD* mutants using ELISA (CDiff Check ™- 60, TechLab Inc). GDH was readily detected in all seven hamsters challenged with JIR8094 strain, but not from the *gluD* mutant challenged hamsters. Student t test was performed and the * indicates *p* value of <0.001
(PDF)

S2 Fig Growth curve of parent and *gluD* mutant strains. Bacterial strains were inoculated and were grown overnight in TY medium with thiamphenicol (15 μg/ml). Then 100 μl of the overnight culture was used to inoculate fresh 10 ml medium and the turbidity of the culture was monitored every 4 hours spectrometrically at OD600nms.
(PDF)

S3 Fig Survival curve of the mixed infection study. Kaplan-Meier survival curve of clindamycin-treated Syrian hamsters inoculated with 2,000 *C*. *difficile* cells (either Parent; or *gluD* mutant; or 1000 Parent+ 1000 *gluD* mutant cells). Animals were monitored every four hours for the symptoms of wet tail, poor fur coat, lethargy, hunch posture and were scored from 1–5. A cumulative score of 12 was assigned as the euthanization point.
(PDF)

S4 Fig Hamster groups used in the cecal amino acid analyses. Schematic diagram of the hamster groups used for the cecal amino acid analyses experiment.
(PDF)

S1 Methods Quantitative PCR (qPCR) analysis of *C*. *difficile* in fecal contents. (PDF)

S1 Table Oligonucleotides used in the study. (PDF)

S2 Table Determining *C*. *difficile* colonization using quantitative PCR with fecal DNA. (PDF)
