# Correction: Importance of Glutamate Dehydrogenase (GDH) in *Clostridium difficile* Colonization *In Vivo*

**DOI:** 10.1371/journal.pone.0165579

**Published:** 2016-10-21

**Authors:** Brintha Parasumanna Girinathan, Sterling Braun, Apoorva Reddy Sirigireddy, Jose Espinola-Lopez, Revathi Govind

The fourth author’s last name is spelled incorrectly. The correct name is: Jose Espinola-Lopez. The correct citation is: Girinathan BP, Braun S, Sirigireddy AR, Espinola-Lopez J, Govind R (2016) Importance of Glutamate Dehydrogenase (GDH) in *Clostridium difficile* Colonization *In Vivo*. PLoS ONE 11(7): e0160107. doi:10.1371/journal.pone.0160107
