# Supplementary material for: Importance of Glutamate Dehydrogenase (GDH) in Clostridium difficile Colonization In Vivo
Source: PLoS One. 2016 Jul 28;11(7):e0160107. doi: 10.1371/journal.pone.0160107 (PMC4965041; doi:10.1371/journal.pone.0160107)

S1 Fig. GDH ELISA

Detecting GDH in the cecal contents of the hamsters infected with either JIR8094 or *gluD* mutants using ELISA (CDiff Check ™- 60, TechLab Inc). GDH was readily detected in all seven hamsters challenged with JIR8094 strain, but not from the *gluD* mutant challenged hamsters. Student t test was performed and the * indicates *p* value of <0.001

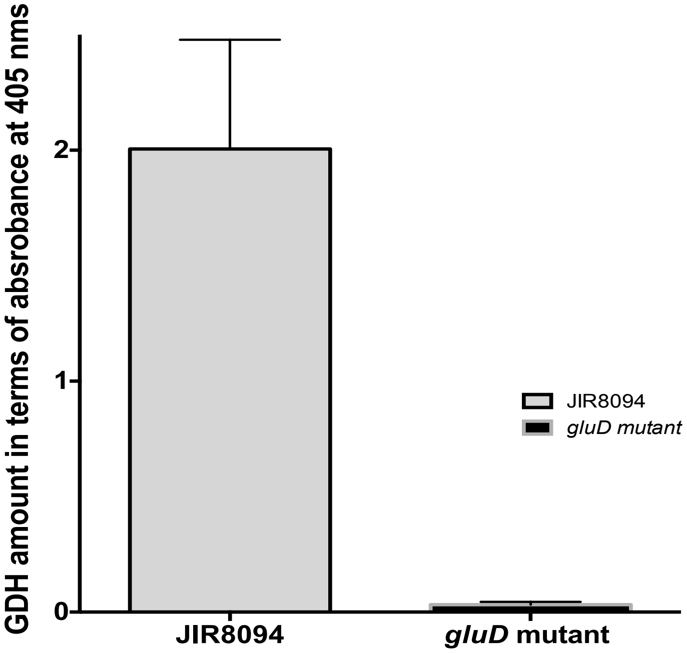

Supplement: S1 Fig — Detecting GDH in the cecal contents of the hamsters infected with either JIR8094 or gluD mutants using ELISA (CDiff Check ™- 60, TechLab Inc). GDH was readily detected in all seven hamsters challenged with JIR8094 strain, but not from the gluD mutant challenged hamsters. Student t test was performed and the * indicates p value of <0.001 (PDF) [file pone.0160107.s001.pdf]
