# Supplementary material for: Importance of Glutamate Dehydrogenase (GDH) in Clostridium difficile Colonization In Vivo
Source: PLoS One. 2016 Jul 28;11(7):e0160107. doi: 10.1371/journal.pone.0160107 (PMC4965041; doi:10.1371/journal.pone.0160107)

S2 Fig. Growth curve of parent and *gluD* mutant strains.

Bacterial strains were inoculated and were grown overnight in TY medium with thiamphenicol (15 µg/ml). Then 100 µl of the overnight culture was used to inoculate fresh 10 ml medium and the turbidity of the culture was monitored every 4 hours spectrometrically at OD600nms.

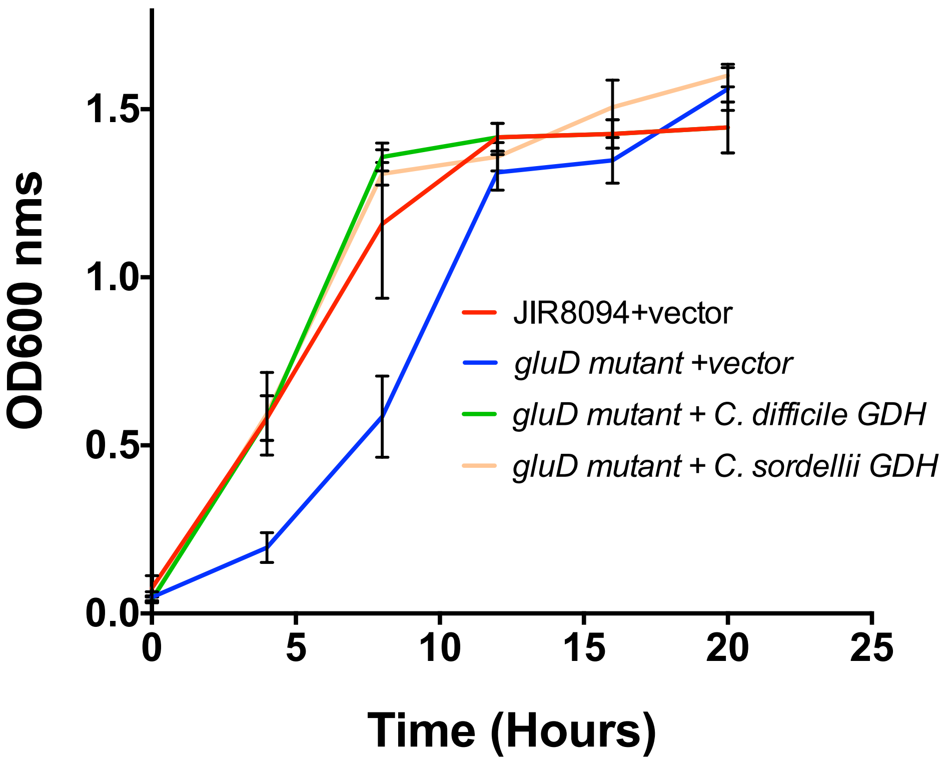

Supplement: S2 Fig — Bacterial strains were inoculated and were grown overnight in TY medium with thiamphenicol (15 μg/ml). Then 100 μl of the overnight culture was used to inoculate fresh 10 ml medium and the turbidity of the culture was monitored every 4 hours spectrometrically at OD600nms. (PDF) [file pone.0160107.s002.pdf]
