# Supplementary material for: Importance of Glutamate Dehydrogenase (GDH) in Clostridium difficile Colonization In Vivo
Source: PLoS One. 2016 Jul 28;11(7):e0160107. doi: 10.1371/journal.pone.0160107 (PMC4965041; doi:10.1371/journal.pone.0160107)
Supplement: S3 Fig — Kaplan-Meier survival curve of clindamycin-treated Syrian hamsters inoculated with 2,000 C. difficile cells (either Parent; or gluD mutant; or 1000 Parent+ 1000 gluD mutant cells). Animals were monitored every four hours for the symptoms of wet tail, poor fur coat, lethargy, hunch posture and were scored from 1–5. A cumulative score of 12 was assigned as the euthanization point. (PDF) [file pone.0160107.s003.pdf]

S3 Fig. Survival curve of the mixed infection study

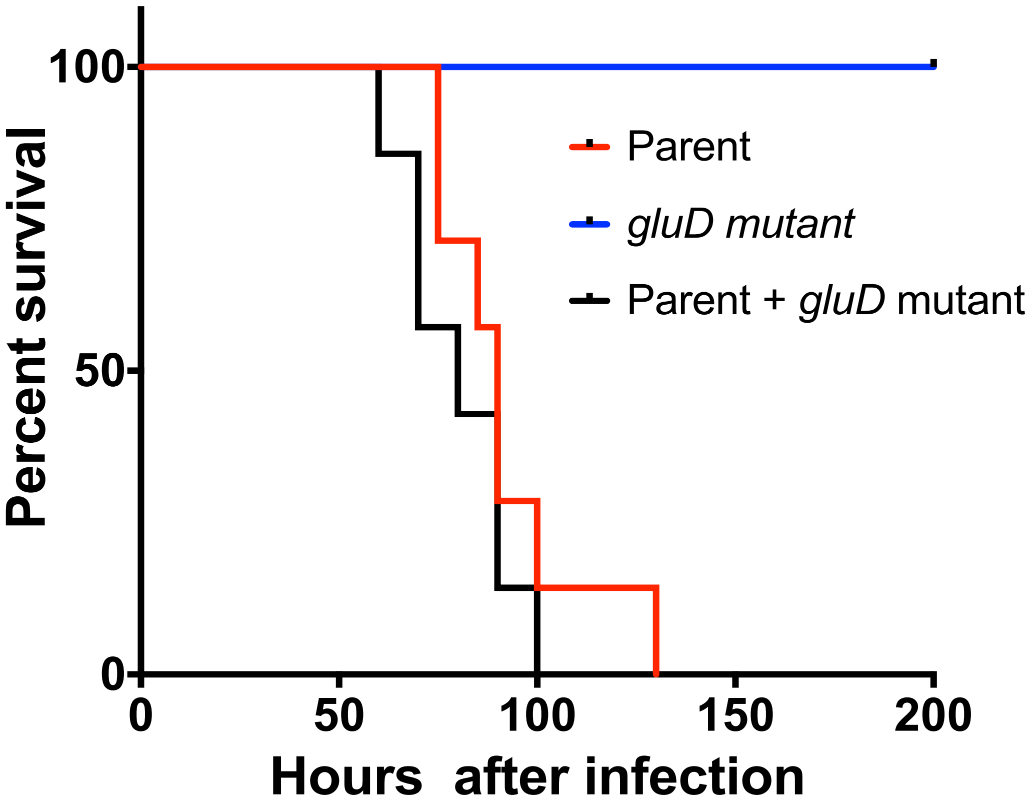

Kaplan-Meier survival curve of clindamycin-treated Syrian hamsters inoculated with 2,000 *C. difficile* cells (either Parent; or *gluD* mutant; or 1000 Parent+ 1000 *gluD* mutant cells). Animals were monitored every four hours for the symptoms of wet tail, poor fur coat, lethargy, hunch posture and were scored from 1-5. A cumulative score of 12 was assigned as the euthanization point.
