# Supplementary material for: Importance of Glutamate Dehydrogenase (GDH) in Clostridium difficile Colonization In Vivo
Source: PLoS One. 2016 Jul 28;11(7):e0160107. doi: 10.1371/journal.pone.0160107 (PMC4965041; doi:10.1371/journal.pone.0160107)
Supplement: S4 Fig — Schematic diagram of the hamster groups used for the cecal amino acid analyses experiment. (PDF) [file pone.0160107.s004.pdf]

S4 Fig. Hamster groups used in the cecal amino acid analyses.

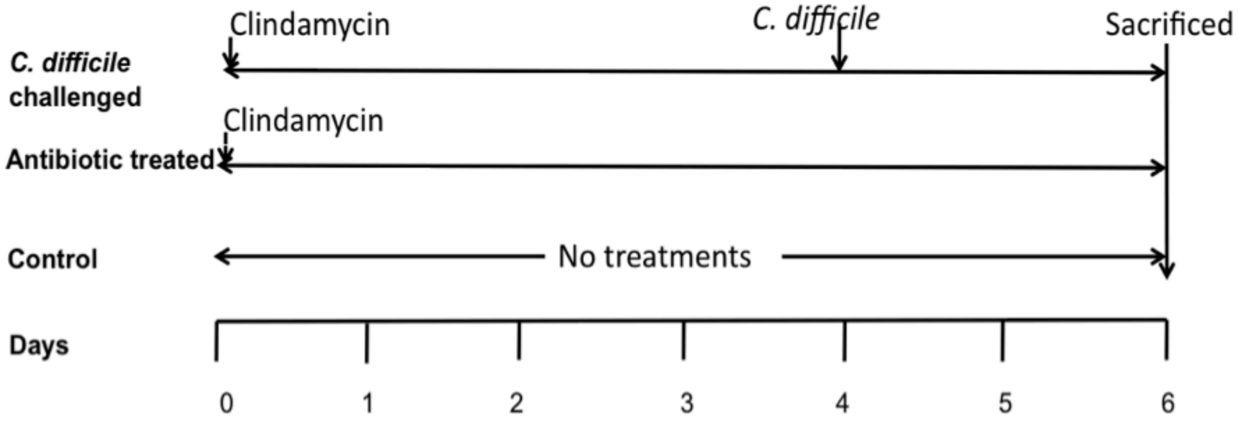

Schematic diagram of the hamster groups used for the cecal amino acid analyses experiment.
