# Supplementary material for: Importance of Glutamate Dehydrogenase (GDH) in Clostridium difficile Colonization In Vivo
Source: PLoS One. 2016 Jul 28;11(7):e0160107. doi: 10.1371/journal.pone.0160107 (PMC4965041; doi:10.1371/journal.pone.0160107)
Supplement: S1 Methods — (PDF) [file pone.0160107.s005.pdf]

Supplemental Materials and Methods:

Quantitative PCR (qPCR) analysis of *C. difficile* in fecal contents

To monitor the *C. difficile* colonization, we started collecting fecal pellets a day after *C. difficile* challenge until the hamsters start developing diarrheal symptoms. Collected fecal materials were stored in -80ºC until they were processed for DNA extraction. One gram of fecal content per hamster was used to prepare bacterial DNA using PowerFecal® DNA Isolation kit (MO BIO Carlsbad, CA, USA). The DNA concentration was measured by Nanodrop and was normalized to 100 ngs/ PCR reaction. The PCR detection of *C. difficile* was performed using previously standardized primers specific for *C. difficile* 16S rRNA. The qPCR was performed using iTaq Universal SYBR Green Supermix (BIORAD, USA). After 40 cycles of PCR amplification, the Ct values were used to evaluate the presence of absence of *C. difficile* in the fecal materials. Ct values and the known number of JIR8094 *C. difficile* cells (counts determined microscopically) were determined and are presented in supplemental table 2B. Ct value of 30 and 28 correlated with ~Log10^3 and Log10^4 bacterial cells per gram of fecal pellets. Similar Ct values were obtained when DNA was isolated from the quantified purified *C. difficile* spores. When the Ct values of the qPCR analysis were above 35 or if the amplicons were not detected, the samples were then regarded as *C. difficile* negative.
