# Supplementary material for: Importance of Glutamate Dehydrogenase (GDH) in Clostridium difficile Colonization In Vivo
Source: PLoS One. 2016 Jul 28;11(7):e0160107. doi: 10.1371/journal.pone.0160107 (PMC4965041; doi:10.1371/journal.pone.0160107)
Supplement: S1 Table — (PDF) [file pone.0160107.s006.pdf]

S1 Table

Oligonucleotides used in the study

Name of the Oligos	Sequence (5' TO 3')	Description
gluDP(F)	AAGCTTCAAGGTTTTAGCTGGGATATCGG	*gluD* promoter with HindIII (FORWARD)
gluDP(R)	TCTAGATGACATTTGAAAGCCCCCTTATAAATA	*gluD* promoter with XbaI (REVERSE)
ORG100	GGT ACC ATG TCA GCA AAG CAA GTC TCG AAA GAT	*rocG* FROM *B. subtilis* (FORWARD) WITH KpnI
ORG101	GAGCTCAATGGTTGTCGGGCCGTTCGCCGCT	*rocG* FROM *B. subtilis* (REVERSE) WITH SacI
ORG 287	GGT ACC TTA AGG GGG CTT TTA AAATGG CTG AA	*C.sordellii gluD* WITH KpnI (FORWARD)
ORG 288	GAGCTCTTAGTGATGAACTAAAGATGGAG	*C. sordellii gluD* WITH SacI (REVERSE)
ORG485	GGATCCAAAGGGGGCATTAAAATGGAAGTAAAAAAATATG GAA	*C. perfringens gluD* with KpnI (FORWARD)
ORG486	GAGCTCTTATATAATACCATGGTCTAACATAGCATCTGCAACC	*C. perfringens gluD* with SacI (REVERSE)
ORG 72	GGTACCATGTCAGGAAAAGATGTAAATGTCTTCGAG	*C. difficile gluD* with KpnI (FORWARD)
ORG 79	GAGCTCTTAATGATGATGATGATGATGGTACCATCCTCTTAATT	*C. difficile gluD with* SacI (REVERSE)
ORG303	ACTAGAGCTCTTAATGTTCTCTCATTGTAACATTG	*C. difficile gluD* with SacI REVERSE (for GDH-20C)
ORG361	GGTACCTATAAGGGGGCTTTCAAATGGAACCAGCAGTTTATGA ATTATTAAAA	*C. difficile gluD* with KpnI (FORWARD For GDH-20N)
RT16S (F)	AAGAGAAGTGTTGTTAAAGTTGATGA	qRT- *C. difficile* 16S rRNA (FORWARD)
RT16S (R)	TCTCCTCCTAAATCACTTAGTTCATAC	qRT-*C. difficile* 16S rRNA (REVERSE)
