# Supplementary material for: Importance of Glutamate Dehydrogenase (GDH) in Clostridium difficile Colonization In Vivo
Source: PLoS One. 2016 Jul 28;11(7):e0160107. doi: 10.1371/journal.pone.0160107 (PMC4965041; doi:10.1371/journal.pone.0160107)
Supplement: S2 Table — (PDF) [file pone.0160107.s007.pdf]

S2 Table: Determining *C. difficile* colonization using quantitative PCR with fecal DNA

A. Monitoring *C. difficile* colonization using quantitative PCR with fecal DNA

Bacterial strains used for hamsters' challenge	Average Ct Values on fecal pellets collected on day			
	0	1	2	3
JIR8094	-	30±0.3	28±1.2	26±1.3
JIR8094::*gluD*	-	-	-	-*
JIR8094+pMTL84151	-	28±1.8	26±1.5	24±1.6 #
JIR8094:: *gluD*+pRGL58 (WT GDH)	-	29±1.9	28±1.2	25±1.5 #
JIR8094+ pRGL315 (*C. sordellii gluD*)	-	-	32±1.3	30±1.8 #

Seven animals per group were used.
* amplification was undetectable till day 15
# Diarrheal symptoms developed after 3 days and the collection of fecal pellets were suspended.
Ct values ± standard error are presented in the table.

B. Table below was generated using Ct values obtained with DNA from known number of cells/spores and were used as standard.

Log_{10} cells/Spores used	Ct values obtained with DNA from vegetative cells	Ct values obtained with DNA from spores
5	25±2.3	24±2.6
4	28±3.1	29±2.1
3	30±2.5	31±1.4
2	33±1.7	32±2.3
1	35±2.1	34±2.5
0	38±2.0	Not Detectable

Genomic DNA was extracted from 10^6, 10^5 and 10^4 cells or spores. We found it difficult to prepare DNA from fewer bacterial cells. Hence, 10 fold serial dilutions of DNA extracted from 10^5 cells were prepared. The second, third and fourth dilutions prepared were assumed to correspond to DNA extracted from 10^3, 10^2 and 10 cell/spores, respectively. Quantitative PCR was performed and the Ct values obtained were listed in the table. The data listed is mean with standard error of the results from triplicate samples.
